# IRAK-2 Regulates IL-1-Mediated Pathogenic Th17 Cell Development in Helminthic Infection

**DOI:** 10.1371/journal.ppat.1002272

**Published:** 2011-10-06

**Authors:** Patrick M. Smith, Berri Jacque, James R. Conner, Alexander Poltorak, Miguel J. Stadecker

**Affiliations:** 1 Immunology Program, Sackler School of Biomedical Sciences, Tufts University School of Medicine, Boston, Massachusetts, United States of America; 2 Department of Pathology, Sackler School of Biomedical Sciences, Tufts University School of Medicine, Boston, Massachusetts, United States of America; Trudeau Institute, United States of America

## Abstract

Infection with the trematode parasite *Schistosoma mansoni* results in distinct heterogeneity of disease severity both in humans and in mice. In the experimental mouse model, severe disease is characterized by pronounced hepatic egg-induced granulomatous inflammation mediated by CD4 Th17 cells, whereas mild disease is associated with reduced hepatic inflammation in a Th2-skewed cytokine environment. Even though the host’s genetic background significantly impacts the clinical outcome of schistosomiasis, specific gene(s) that contribute to disease severity remain elusive. We investigated the schistosome infection in wild-derived mice, which possess a more diverse gene pool than classically inbred mouse strains and thus makes them more likely to reveal novel mechanisms of immune regulation. We now show that inbred wild-derived MOLF mice develop severe hepatic inflammation with high levels of IL-17. Congenic mice with a MOLF locus in chromosome 6, designated Why1, revealed high pathology and enabled the identification of *Irak2* as the pathogenic gene. Although IRAK-2 is classically associated with TLR signaling, adoptive transfer of CD4 T cells revealed that IRAK-2 mediates pathology in a CD4 T cell specific manner by promoting Th17 cell development through enhancement of IL-1β-induced activation of transcription factors RORγt and BATF. The use of wild-derived mice unravels IRAK-2 as a novel regulator of IL-1-induced pathogenic Th17 cells in schistosomiasis, which likely has wide-ranging implications for other chronic inflammatory and autoimmune diseases.

## Author Summary

Schistosomes are trematode helminths that cause widespread disease in vertebrates and are responsible for over 200 million human infections worldwide. The species *Schistosoma mansoni* causes a hepatic granulomatous inflammatory and fibrosing reaction against tissue trapped parasite eggs that varies greatly in humans and among mouse strains, implying that the host’s genetic background plays a critical role in determining disease severity. Although exacerbated hepatic inflammation is known to be associated with an increase in CD4 Th17 cells, specific genes conducive to high pathology are unknown. In this study we used genetically diverse inbred wild-derived mice and found that their natural severe immunopathology and high IL-17 levels are regulated by the interleukin-1 (IL-1) receptor-associated kinase-like 2 (IRAK-2). We demonstrate that T cell intrinsic IRAK-2 affects disease severity by enhancing the development of Th17 cells, which results from an increased sensitivity to IL-1β induced activation of the lineage-specific transcription factors RORγt and BATF. Our findings thus identify IRAK-2 as a single regulator of pathogenic Th17 cell development in murine schistosomiasis and reveal a novel mechanism that is likely to operate in other chronic inflammatory and autoimmune diseases.

## Introduction

The genetic analysis of complex traits has been critical to our understanding of the molecular mechanisms that underlie disease processes. Quantitative trait loci (QTL) are genomic intervals, whose variation is responsible for the majority of genetic diversity in human disease susceptibility and severity. As a model of human genetics, classical inbred mouse strains have been instrumental in identifying QTL. Murine schistosomiasis represents an extensively characterized model of a major human infectious disease that shares similar mechanistic features with many autoimmune and chronic inflammatory diseases [1], [2]. Although several QTL underlying pathology in schistosomiasis have been identified to date, mouse genetic studies have not entirely recapitulated the genetic complexity that is likely to determine the disease course in humans. One reason for this is the relatively limited genetic diversity among classically inbred strains. These mice are derived from a restricted number of founder animals predominantly within the *Mus mus domesticus* subspecies and therefore do not reach the level of diversity observed in humans [3], [4]. We reasoned that this limited diversity was a major problem that has made it difficult to identify genes that underlie even well defined traits, leaving a compelling need for new models of genetic analysis.

Wild-derived inbred mice diverged from a common ancestor with classical strains more than one million years ago. As a result of this early divergence, many of the wild-derived strains have large genomic regions originating from the subspecies *M. m. musculus* and *M. m. castaneus* [3], [4], which provides them with a unique and more genetically diverse gene pool compared with classically inbred strains. The genetic diversity of wild-derived mice resembles that seen in humans, which makes them more suitable for the analysis of complex traits, such as host-pathogen interactions. Furthermore, novel phenotypes identified in wild-derived mice are likely to have increased biological relevance given that they have arisen in an evolutionarily driven context[5]. Wild-derived mice have proved useful as genetic models in identifying novel phenotypic variants in studies exploring host responses to infection with pathogens, such as *Salmonella typhimurium* [6], as well as identifying several loci that confer resistance to TNFα induced toxic shock[7].

The main pathology in murine schistosomiasis consists of a granulomatous inflammatory and fibrosing reaction in the liver and intestine against tissue trapped parasite eggs, which is a host adaptive immune response mediated by CD4 T cells specific for schistosome egg antigens (SEA)[1], [8], [9]. Following infection with *S. mansoni*, most humans develop mild, “intestinal schistosomiasis”, however 5-10% develop a severe inflammatory and fibrosing response, which leads to a potentially lethal form of the disease, “hepatosplenic schistosomiasis”[10]. This variation also exists in a mouse model of schistosomiasis, where CBA mice develop pronounced hepatic granulomatous inflammation, while C57BL/6 (BL/6) mice develop significantly smaller granulomas with milder hepatic inflammation[11], [12].

Th17 cells represent a unique lineage of T cells that act as potent proinflammatory mediators and have been shown to play a significant role in a number of inflammatory and autoimmune diseases, such as experimental autoimmune encephalomyelitis (EAE), collagen-induced arthritis, psoriasis and inflammatory bowel disease[13], [14], [15], [16]. Th17 cells are characterized by their expression of the transcription factor RORγt[17] as well as by their requirement of IL-6, TGF-β, IL-23 and IL-21 to differentiate and expand[18], [19], [20], [21], [22], [23]. IL-1β is also of particular importance in Th17 cell differentiation and pathogenesis [24], [25], [26], [27]. In schistosomiasis, IL-23 and IL-1β are necessary for IL-17 production in response to parasite eggs and severe immunopathology correlates with increased production of IL-17[28], [29], [30]. Furthermore, *in vivo* neutralization of IL-17 significantly reduces the immunopathology[12].

In an attempt to identify novel mechanisms that govern severe disease, we assessed the schistosome infection in wild-derived inbred mice of the MOLF strain. We previously have shown that in MOLF mice, TLR ligation in macrophages *in vitro* leads to significantly higher transcription of proinflammatory cytokines than in classically inbred BL/6 mice [31]. To examine if their bias towards a proinflammatory response also occurs in an *in vivo* infection model, we infected MOLF mice with *S. mansoni* and found that they develop severe liver immunopathology associated with a high Th17 response. Although we anticipated identifying novel factors that regulate T cell function via APC responses, we were surprised to find a T cell-intrinsic regulator of IL-17 production. We now demonstrate that a single gene, *Irak2*, is capable of controlling severe pathology in murine schistosomiasis. Furthermore, we provide evidence of a novel role for IRAK-2 in IL-1β-mediated pathogenic Th17 cell development. Our study also demonstrates the utility of wild-derived mice as a model to identify novel gene networks and further refine our understanding of immune signaling pathways.

## Results

### MOLF mice develop severe immunopathology with high levels of IL-17 following schistosome infection

**Figure 1:**
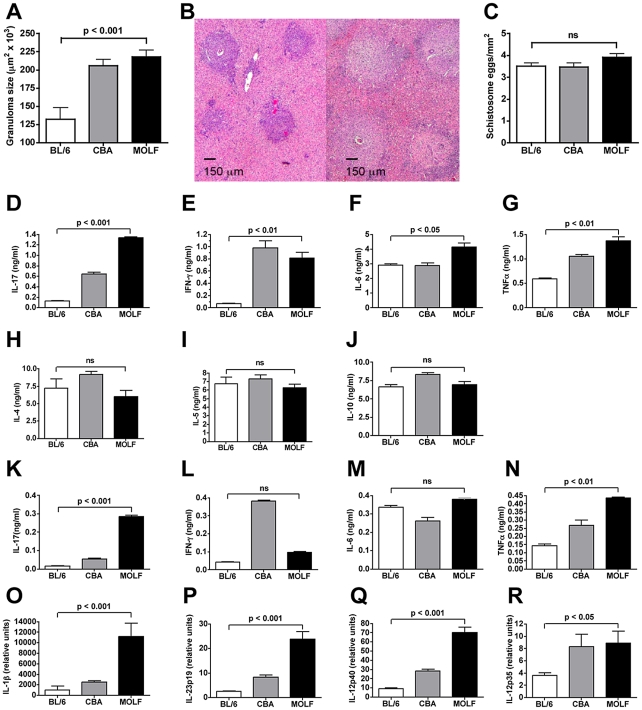
Schistosome-infected wild-derived MOLF mice develop severe egg-induced immunopathology and a proinflammatory cytokine profile. After a 7-week infection, BL/6, CBA and MOLF mice were analyzed for hepatic immunopathology and cytokine expression. (**A**) Granuloma size was measured by morphometric analysis. At least 15 granulomas were measured per liver in 5 individual mice per group. Error bars represent the mean +/- SD of granuloma size within a group. **B**) H & E stain of representative hepatic egg granulomas from BL/6 (left panel) and MOLF mice (right panel). (**C**) Mean +/- SD of number of *S. mansoni* eggs in 1 mm^2^ fields on H/E stained liver sections. 10–20 fields were counted per liver section and 10–15 livers were counted per mouse group. MLNC production of IL-17, IFN- γ, IL-6 and TNFα (**D-G**) and IL-4, IL-5 and IL-10 (**H-J**), and GC production of IL-17, IFN-γ, IL-6 and TNFα (**K-N**) following stimulation with 15 µg/ml of SEA for 48 hours, as measured by ELISA. Expression of IL-1β, IL-23p19, IL-12p40 and IL-12p35 (**O-R**) was measured by real-time quantitative RT-PCR. RNA was isolated from the livers of infected mice and data were normalized to GAPDH. Error bars represent means of triplicate determinations +/- SD. Results are representative of at least 4 independent experiments with at least 5 mice per group. P values were determined by one-way ANOVA. ns  =  not significant.

Seven weeks after infection with 85 cercariae of *S. mansoni*, MOLF mice were debilitated, as defined by hunched posture, reduced activity and scruffy appearance, and exhibited significantly enhanced hepatic egg-induced immunopathology when compared with BL/6 mice, with granulomatous lesions in some instances larger than those seen in the high-pathology control CBA mice (**Fig. 1A**). Individual granulomas from MOLF mice consisted of significantly larger perioval aggregates of macrophages/histiocytes and lymphocytes admixed with eosinophils and neutrophils, with a greater tendency to infiltrate the surrounding hepatic parenchyma than in BL/6 mice (**Fig. 1B**). However, the number of schistosome eggs present in the livers did not significantly differ between the mouse groups, indicating that the parasite load did not correlate with the extent of pathology (**Fig. 1C**). Analysis of antigen specific cytokine production by schistosome egg antigen (SEA)-stimulated draining mesenteric lymph node (MLN) cells (MLNC) from infected mice revealed that MOLF mice produced strikingly higher amounts of IL-17 than BL/6 and even CBA mice (**Fig. 1D**). There also was a significant, but less pronounced, increase in IFN-γ, IL-6 and TNFα (**Fig. 1E–G**). However, there were no significant differences in IL-4, IL-5 or IL-10 between BL/6, CBA and MOLF mice (**Fig. 1H–J**).

Cytokine production in MLNC typically correlates with that produced in the affected liver. In order to confirm this in MOLF mice, we isolated granuloma cells (GC) and analyzed their specific response to SEA. Similar to the MLNC, MOLF GC produced very high amounts of IL-17 compared with both BL/6 and CBA mice (**Fig. 1K**). There also was higher IFN-γ, IL-6 and TNFα production in MOLF vs. BL/6 mice, but only in the case of TNFα was the difference statistically significant (**Fig. 1L–N**). There were no significant differences in IL-4, IL-5 or IL-10 (data not shown). Analysis of cytokines involved in the development of Th17 cells revealed that IL-1β, as well as IL-23p19 and IL-12p40, the subunits that make up IL-23, were expressed at much higher levels in the livers of MOLF mice than BL/6 and CBA mice (**Fig. 1O–Q**), whereas the difference in the IL-12-specific subunit IL-12p35 was less striking (**Fig. 1R**). These results demonstrate that wild-derived MOLF mice produce exceedingly high levels of Th17-related cytokines, suggesting a potentially novel mechanism of severe immunopathology.

### Severe immunopathology and increased IL-17 production are controlled by the MOLF allele of the *Why1* locus

**Figure 2:**
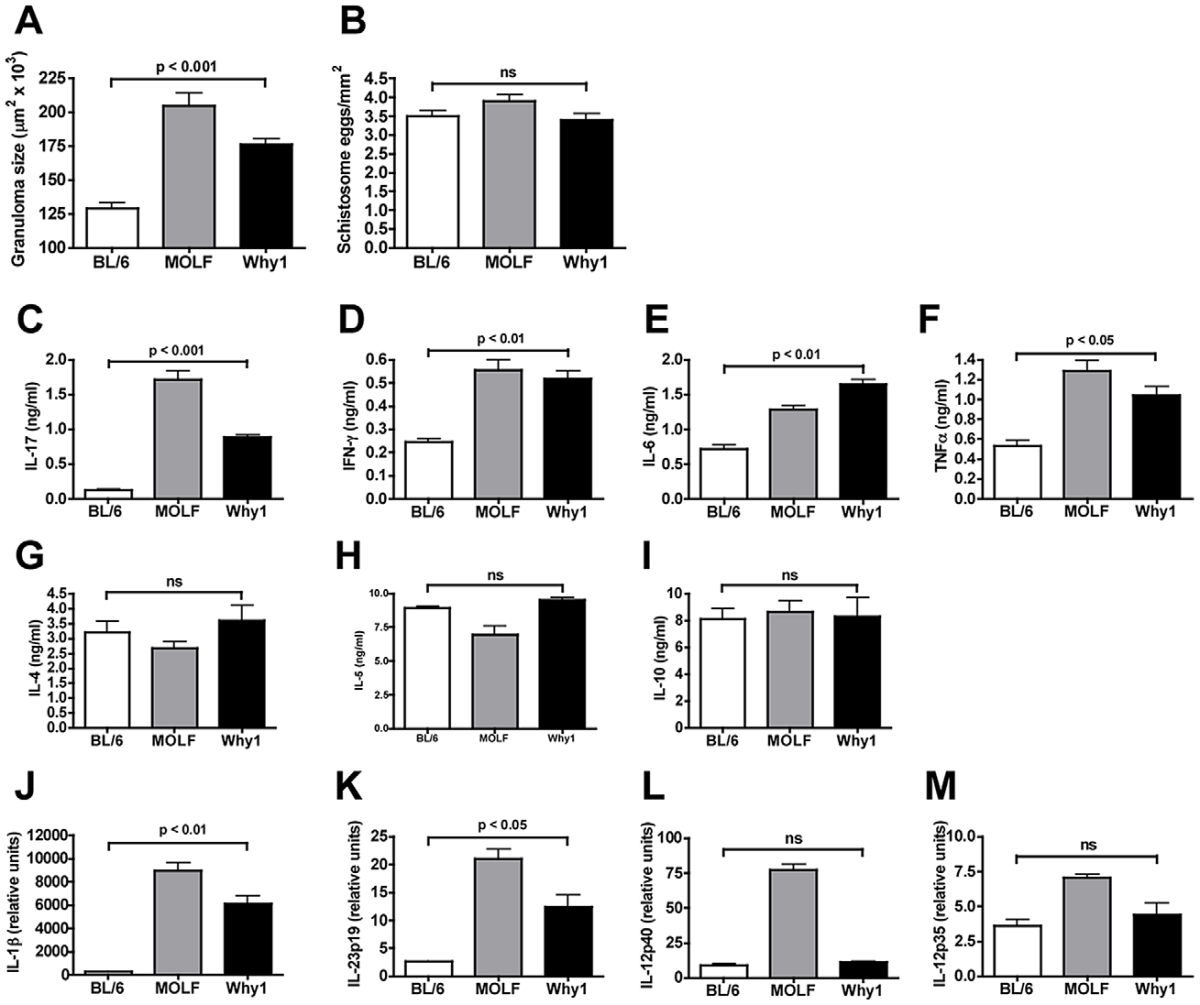
Severe egg-induced immunopathology and proinflammatory cytokine production are controlled by the Why1 locus. After a 7-week infection, BL/6, MOLF and Why1 mice were analyzed for hepatic immunopathology and cytokine expression. (**A**) Granuloma size was measured by morphometric analysis. At least 15 granulomas were measured per liver in 5 individual mice per group. Error bars represent the mean +/- SD of granuloma size within a group. (**B**) Mean +/- SD of number of *S. mansoni* eggs in 1 mm^2^ fields on H/E stained liver sections. 10–20 fields were counted per liver section and 10–15 livers were counted per mouse group. MLNC production of IL-17, IFN- γ, IL-6 and TNFα (**C-F**) and IL-4, IL-5 and IL-10 (**G-I**) following stimulation with 15 µg/ml of SEA for 48 hours, as measured by ELISA. Expression of IL-1β, IL-23p19, IL-12p40 and IL-12p35 (**J-M**) was measured by real-time quantitative RT-PCR. RNA was isolated from the livers of infected mice and data were normalized to GAPDH. Error bars represent means of triplicate determinations +/- SD. Results are representative of at least 3 independent experiments with at least 5 mice per group. P values were determined by one-way ANOVA. ns  =  not significant.

We previously mapped the IL-6 hyper-responsiveness of MOLF macrophages to TLR stimulation to a dominant locus on chromosome 6, designated *Why1* [32]. Since MOLF mice reacted to schistosome infection with an overwhelmingly proinflammatory response, we postulated that the *Why1* locus may also play a role in this phenotype in the context of live infection. To assess the effect of the *Why1* locus directly, we used congenic mice (Why1 mice), which contain the MOLF allele of the *Why1* locus on a BL/6 background. After a 7-week schistosome infection, Why1 mice appeared debilitated, as defined above, and displayed a significant increase in hepatic granulomatous inflammation compared with BL/6 mice, although not to the same extent as MOLF mice (**Fig. 2A**), with the differences not attributable to dissimilar egg burdens (**Fig. 2B**). There was significantly greater IL-17, IFN-γ, IL-6 and TNFα production by SEA-stimulated MLNC in Why1 vs. BL/6 mice (**Fig. 2C–F**), although IL-4, IL-5 or IL-10 were not significantly different (**Fig. 2G–I**). Expression of IL-1β and IL-23p19 (**Fig. 2J,K**), but not of IL-12p40 or IL-12p35 (**Fig. 2L,M**), was also higher in Why1 vs. BL/6 mice. These findings demonstrate that Why1 mice largely recapitulate the pathology and IL-17 secretion seen in MOLF mice, and identify Why1 as a key locus associated with increased egg-induced immunopathology and Th17 cell development.

### Severe immunopathology and increased IL-17 production controlled by the *Why1* locus are mediated by CD4 T cells

**Figure 3:**
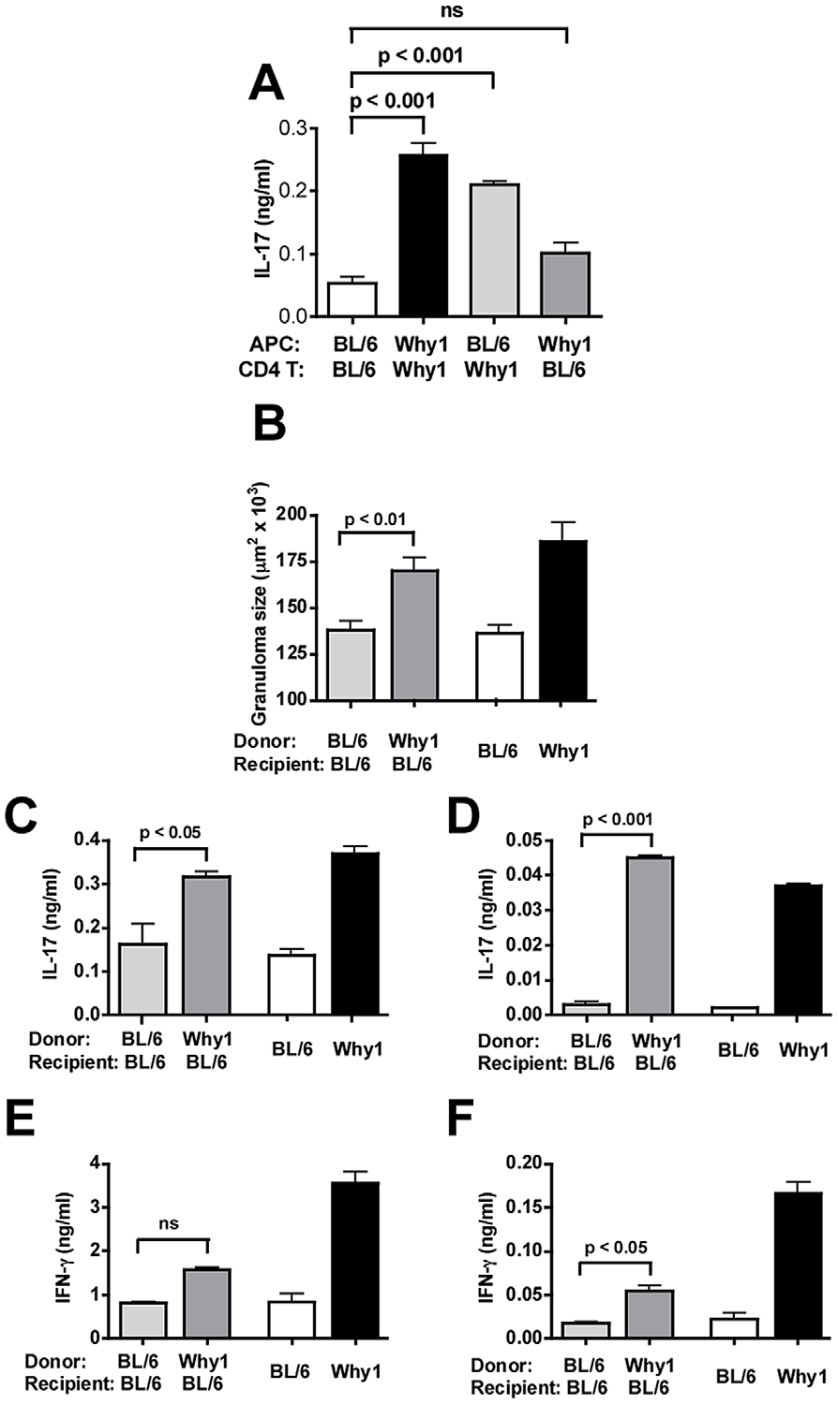
Why1-dependent severe egg-induced immunopathology is mediated by CD4 T cells. (**A**) The indicated combinations of CD4 T cells from 7 week-infected mice plus normal irradiated splenic APC were stimulated with SEA for 48 hours, and IL-17 production was measured by ELISA. (**B-F**) Purified naïve splenic BL/6 or Why1 CD4 T cells (8×10^6^) were injected i.v. into sublethally irradiated BL/6 recipient mice, which were subsequently infected with *S. mansoni*. Infected BL/6 and Why1 mice are shown for comparison. (**B**) Granuloma size in recipient mice was measured by morphometric analysis. IL-17 (**C-D**) and IFN-γ (**E-F**) production by bulk MLNC (**C,E**) and purified MLN CD4 T cells plus irradiated splenic APC (**D,F**) stimulated with SEA for 48 hours, was measured by ELISA. Error bars represent means of triplicate determinations +/- SD. Results are representative of two (**A**) or three (**B-F**) independent experiments with at least 4 mice per group. P values were determined by one-way ANOVA. ns  =  not significant.

The *Why1* locus contains >200 possible causal genes that could underlie pathology in a complex trait such as the response to schistosome infection. We therefore sought to reduce the number of possible candidate genes by further defining the phenotype of Why1 mice. Based on previous mapping of the *Why1* locus in macrophages[31], [32], we hypothesized that severe disease and increased IL-17 production were induced by innate immune cells. To this effect, in order to avoid bias towards any one particular APC type, we used an *in vitro* system involving bulk naïve splenic APC together with CD4 T cells isolated from MLN of infected mice. SEA-stimulated Why1 APC-CD4 T cell cocultures produced markedly higher levels of IL-17 than BL/6 controls (**Fig. 3A**). However, surprisingly, Why1 T cells in combination with BL/6 APC resulted in higher IL-17 production than Why1 APC in combination with BL/6 T cells, which did not significantly differ from the IL-17 produced by the all-BL/6 coculture; moreover, Why1 T cells in combination with either Why1 or BL/6 APC resulted in high IL-17 production (**Fig. 3A**). These findings suggest that CD4 T cells play a decisive role in dictating the levels of IL-17; however, since the Why1 T cells were isolated from infected Why1 mice, an influence of Why1 APC on impending Th17 cell development could not be excluded. We therefore adoptively transferred *naïve* splenic CD4 T cells from uninfected Why1 mice to allow antigen specific Th17 cell differentiation to take place *in vivo* in the absence of Why1 APC. As shown in **Fig. 3B**, transfer of Why1 T cells caused a sharp increase in granulomatous inflammation in infected BL/6 recipients, whereas a similar transfer of BL/6 T cells had no effect. Furthermore, SEA stimulated bulk MLNC (**Fig. 3C**), or MLN CD4 T cells (**Fig. 3D**), from infected BL/6 mice recipients of Why1 T cells produced significantly more IL-17 than those receiving BL/6 T cells. There was also an increase in IFN-γ, although to a lesser extent than IL-17 (**Fig. 3E,F**). These results indicate that the *Why1* locus controls severe immunopathology and enhances Th17 cell development via a CD4 T cell-mediated mechanism.

### IRAK-2 regulates IL-17 production by CD4 T cells

**Figure 4:**
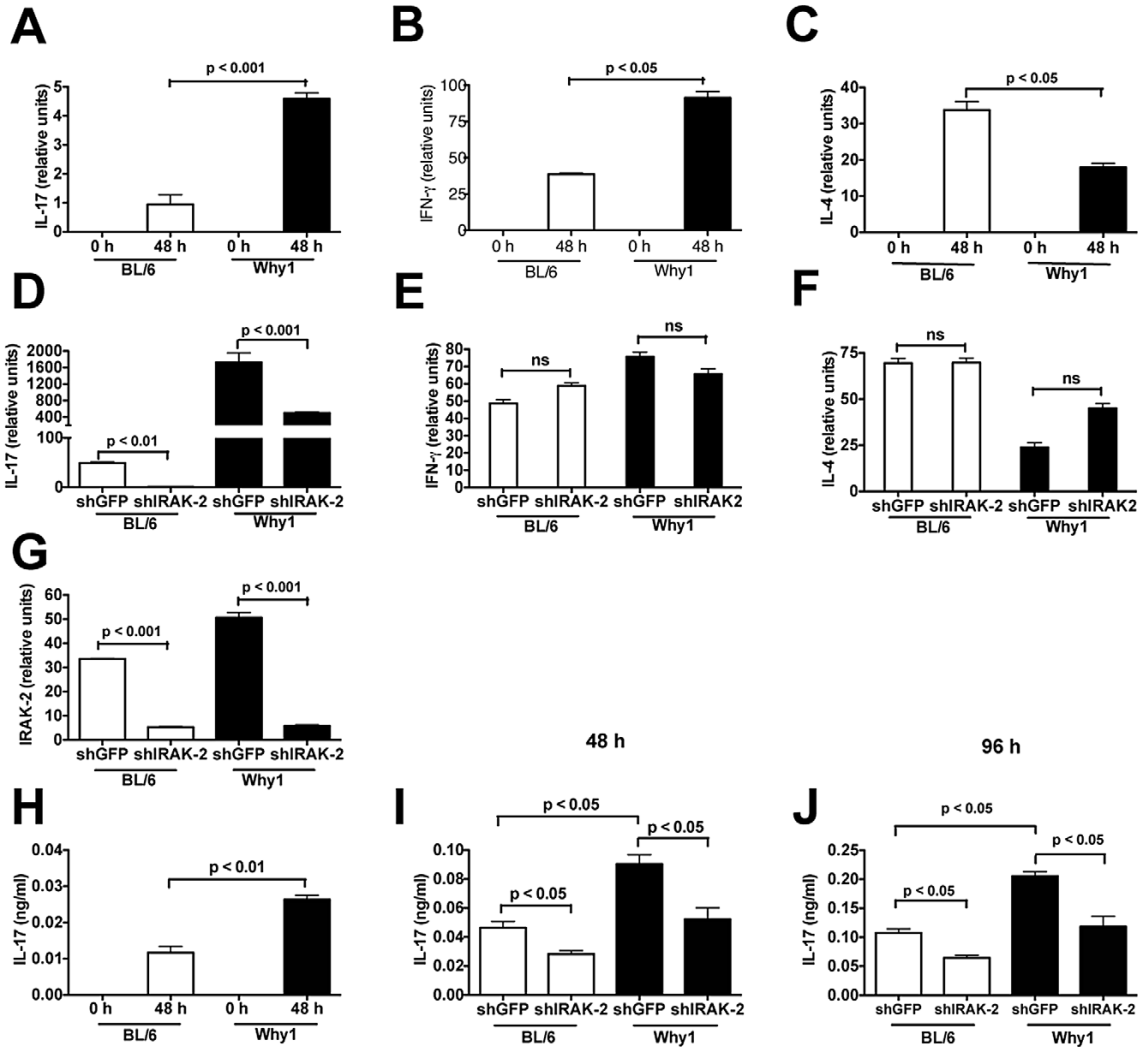
IRAK-2 is necessary for CD4 T cell-specific IL-17 production. (**A-C**) IL-17, IFN-γ and IL-4 mRNA expression by naïve CD4 T cells from BL/6 and Why1 mice, either unstimulated (0 h), or stimulated with anti-CD3/CD28 for 48 hours, as measured by quantitative real-time PCR. (**D-F**) BL/6 and Why1 CD4 T cells were first treated with shRNA against IRAK-2 or control GFP and then stimulated with anti-CD3/CD28 for 48 hours. IL-17, IFN-γ and IL-4 mRNA expression was measured by quantitative real-time PCR. (**G**) To assess knockdown efficiency, mRNA was harvested and cDNA was amplified with IRAK-2 or GAPDH specific primers, and expression was measured by quantitative real-time PCR. (**H**) IL-17 production by naive CD4 T cells from BL/6 and Why1 mice, either unstimulated (0 h), or stimulated with anti-CD3/CD28 for 48 hours, as measured by ELISA. (**I-J**). IL-17 production in BL/6 and Why1 CD4 T cells pretreated with shRNA against IRAK-2 or control GFP and then stimulated with anti-CD3/CD28 for 48 and 96 hours, as measured by ELISA. Error bars represent means of triplicate determinations +/- SD from one of three independent experiments with similar results. P values were determined by one-way ANOVA and Students *t* test. ns  =  not significant.

Why1 CD4 T cells confer enhanced immunopathology and IL-17 production to infected recipient BL/6 mice, however, the cytokine responses of Why1 T cells in the absence of schistosome infection are unknown. To determine if an inherent proinflammatory bias exists, Why1 and BL/6 CD4 T cells were isolated by negative selection from the spleens of naïve uninfected mice and stimulated with anti-CD3/CD28. Both displayed similar basal levels of cytokine mRNA, however, there were significantly higher levels of IL-17 and IFN-γ in stimulated Why1 T cells than in BL/6 T cells (**Fig. 4A,B**), while levels of IL-4 were higher in BL/6 T cells than in Why1 T cells (**Fig. 4C**), suggesting that Why1 CD4 T cells are biased towards a Th17/Th1 proinflammatory phenotype.

Using shRNA knockdown technology, we previously demonstrated that *Irak2* is the gene responsible for the enhanced proinflammatory response of Why1-derived macrophages following TLR stimulation [31]. This observation supported the notion that *Irak2* is primarily involved in innate immune response signaling. To investigate whether there is a direct involvement of IRAK2 in up-regulation of inflammatory cytokines in activated Why1 T cells, we employed shRNA technology to examine the effect of IRAK-2 knockdown on levels of cytokine mRNA. We observed a significant reduction in IL-17 mRNA induced by IRAK-2-specific (but not by control shGFP) hairpin treatment in Why1 T cells (**Fig. 4D**). Knockdown of IRAK-2 also suppressed IL-17 by BL/6 T cells (**Fig. 4D**), indicating that its effect is not specific to MOLF mice. Infection with the IRAK-2-specific hairpin led to a specific decrease in IL-17 expression, which strongly suggests that IRAK-2 is involved in transcriptional up-regulation of IL-17. Interestingly, there was no significant effect on IFN-γ (**Fig. 4E**) or IL-4 (**Fig. 4F**). Knockdown of IRAK-2 in BL/6 and Why1 T cells was confirmed by mRNA analysis due to the lack of a suitable antibody against IRAK-2 (**Fig. 4G**).

To confirm these results, we measured the effect of IRAK-2 on IL-17 protein levels. Why1 T cells stimulated with anti-CD3/CD28 produced significantly more IL-17 than their BL/6 counterparts (**Fig. 4H**). Furthermore, knockdown of IRAK-2 significantly decreased IL-17 production in both Why1 and BL/6 T cells at 48 and 96 hours following stimulation, indicating that this effect was stable over time (**Fig. 4I, J**). Unstimulated cells produced no detectable cytokine at either time point, and knockdown of IRAK-2 did not significantly affect IFN-γ or IL-4 protein levels (data not shown). IFN-γ, unlike IL-17, is largely dependent on the JAK1/STAT1 activation pathway, whereas IL-17’s promoter has well characterized consensus sites for the NFAT and NF-κB transcription factors [33], [34], [35]. We thus postulate that the specific effect of IRAK-2 on IL-17 is related to its role in activating NF-κB and p38 MAP kinase. These results uncover a novel role for IRAK-2 in directing Th17 cell polarization.

### IRAK-2 determines the severity of the schistosome infection *in vivo*

**Figure 5:**
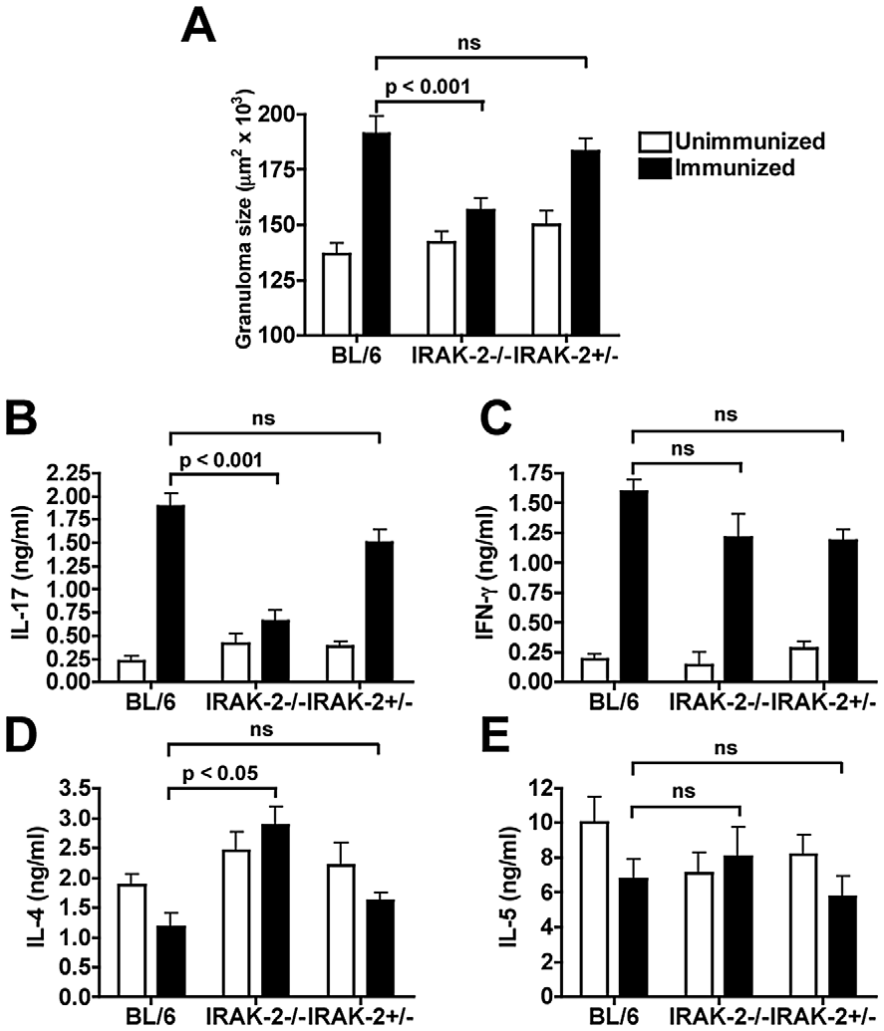
IRAK-2 mediates severe egg-induced immunopathology and CD4 T cell IL-17 production. IRAK-2-/-, littermate IRAK-2-/+ and BL/6 mice were, or were not, immunized s.q. with 50 µg of SEA/CFA prior and following infection, as previously described. (**A**) Granuloma size was measured by morphometric analysis after 7 weeks of infection. At least 30 granulomas were measured per liver in 5-7 individual mice per group. Error bars represent the mean +/- SD of granuloma size within a group. (**B**) IL-17, (**C**) IFN-γ, (**D**) IL-4 and (**E**) IL-5 production by CD4 MLN T cells plus irradiated splenic APC, stimulated with SEA for 48 hours, was measured by ELISA. Error bars represent means of triplicate determinations +/- SD. Results are representative of 2 independent experiments. P values were determined by one-way ANOVA. ns  =  not significant.

To directly assess the effect of IRAK-2 on the schistosome infection *in vivo*, we examined the immunopathology and cytokine profile in IRAK-2-deficient (IRAK-2-/-) mice. Since naturally high-pathology IRAK-2-/- mice are currently not available, we took advantage of a model in which severe egg-induced immunopathology with high IL-17 levels can be induced in infected BL/6 mice by immunization with SEA in CFA (SEA/CFA) [12]. This model shares many attributes with naturally occurring high pathology, and has been instrumental in identifying mechanisms controlling the elevated Th17 responses[12], [30], [36], [37]. After 7 weeks of infection, the SEA/CFA-immunized IRAK-2-/- mice appeared healthier and exhibited significantly reduced granulomatous inflammation when compared to similarly treated WT BL/6 mice and IRAK-2+/- littermate controls (**Fig. 5A**). SEA-stimulated MLN CD4 T cells from infected, SEA/CFA-immunized IRAK-2-/- mice also produced significantly less IL-17 than BL/6 and IRAK-2+/- controls, and barely above the levels of their unimmunized counterparts (**Fig. 5B**). Interestingly, while IRAK-2 did not significantly influence the levels of IFN-γ or IL-5 (**Fig. 5C,E**), its absence led to higher IL-4 production (**Fig. 5D**). These results demonstrate that IRAK-2 plays a key role in the development of severe immunopathology and IL-17 production in schistosomiasis.

### IRAK-2 enhances IL-1-induced Th17 cell responses

IL-1β and IL-23 have been shown to play key roles in pathogenic Th17 cell development from naive precursors [26], [27], [38]. We have also demonstrated these cytokines to be necessary for Th17 cell differentiation in high-pathology CBA mice [28]. IRAK-2 plays a key role in MyD88 dependent TLR signaling, however, it also can function downstream of the IL-1 receptor [39], [40]. Our results suggest that the role of IRAK-2 in Th17 cell development is T cell intrinsic (**Figure 3B–F**). To address the molecular basis by which wild-derived IRAK-2 leads to enhanced IL-17 production, we stimulated naïve CD4 T cells with IL-1 and IL-23, alone or in combination, and found that IL-1 *per se* induced IL-17 production only in CD4 T cells from Why1, but not from BL/6 or IRAK-2-/- mice (**Figure 3B–F**); IL-23 alone was ineffective. Since IL-23 is known to induce IL-17 production in memory Th17 cells [41], [42] these results suggest that neither Why1 nor BL/6 T cells had a pre-existing memory phenotype. Rather, IL-23 synergized with IL-1 for a significantly greater IL-17 production by Why1 than either BL/6 or IRAK-2-/- cells (**Fig. 6A**). In CD4 T cells additionally stimulated non-specifically with anti-CD3/CD28, IL-1 again elicited significantly more IL-17 production in Why1 than in BL/6 cells, whereas the lowest levels of IL-17 were produced by T cells from IRAK-2-/- mice (**Fig. 6B**). IL-23 markedly enhanced IL-1-induced IL-17 production by Why1 and BL/6 T cells, but significantly less so in IRAK-2-/- T cells (**Fig. 6C**). These results demonstrate that IL-1-induced IL-17 production by T cells is highly dependent on IRAK-2; they also indicate that IL-23 by itself is unable to stimulate IL-17 production, but significantly potentiates IL-1 in carrying out this function.

**Figure:**
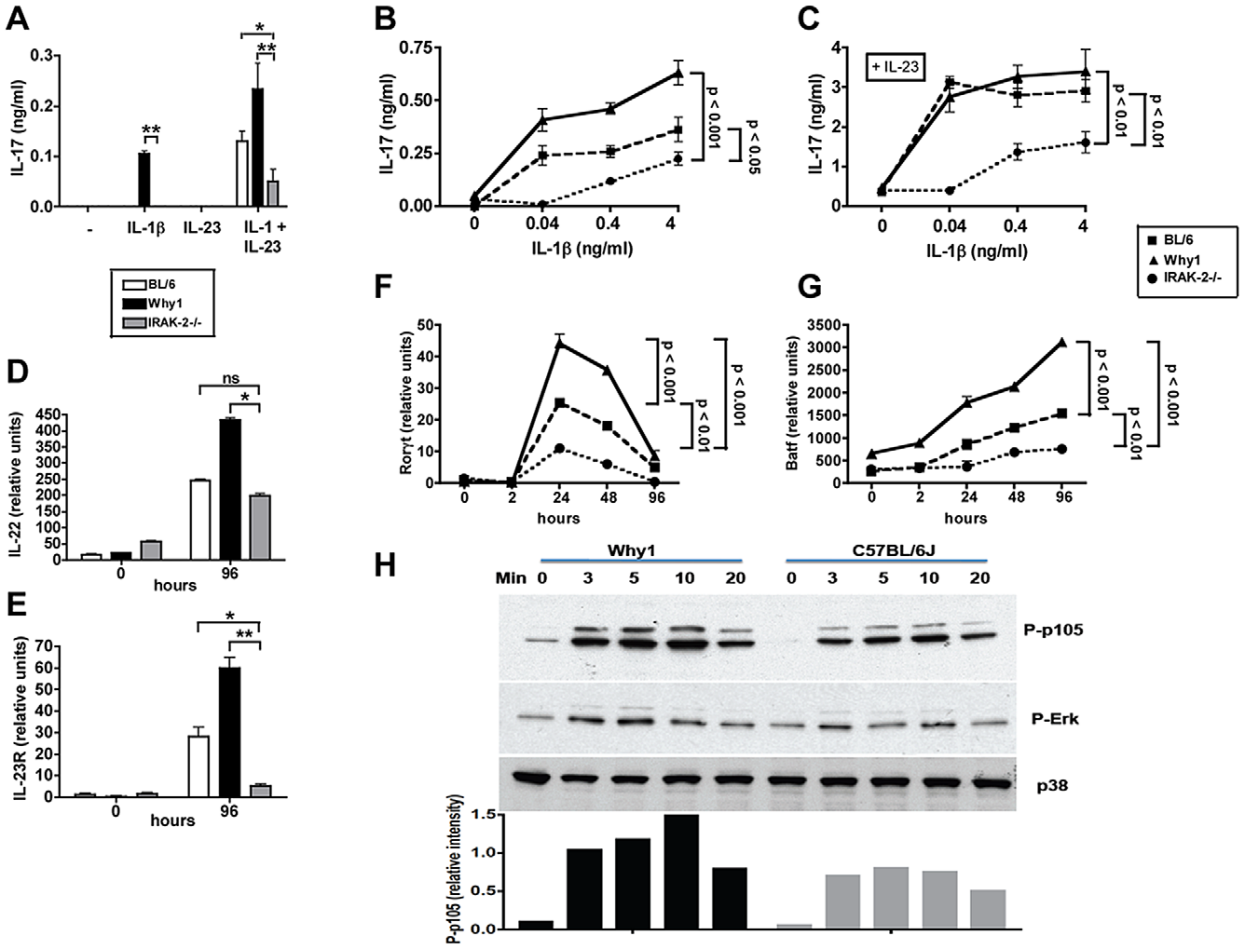
IRAK-2 promotes CD4 T cell IL-17 production and Th17 lineage commitment and activation. (**A**) Naïve splenic CD4 T cells from BL/6, Why1 and IRAK-2-/- mice were stimulated with 4 ng/ml of IL-1β, or 20 ng/ml of IL-23, or both, for 96 hours, and IL-17 production was measured by ELISA. Naïve splenic CD4 T cells were stimulated with anti-CD3/CD28 plus IL-1β, at indicated concentrations, either (**B**) alone or (**C**) in the presence of 20 ng/ml of IL-23 for 96 hours, and IL-17 production was measured by ELISA. Naïve splenic CD4 T cells were stimulated with anti-CD3/CD28 plus 0.4 ng/ml of IL-1β, and (**D**) IL-22, (**E**) IL-23R, (**F**) Rorγt and (**G**) BATF expression were measured by quantitative real-time PCR analysis at the indicated time points. Data were normalized to GAPDH. Error bars represent means of triplicate determinations +/- SD. (**H**) BL/6 and Why1 CD4 T cells were stimulated with 4 ng/ml of IL-1β for the indicated times and cytoplasmic protein lysates were analyzed by Western blot for MAPK (P-Erk) and NF-κB (P-p105) pathway activity. Unphosphorylated p38 kinase was used as a loading control. Densitometric analysis of band intensities was determined for the phospho-p105 blot, in which each band was normalized to its -p38 counterpart and the relative intensities were calculated. Data are representative of 3 (A-G) or 2 (H) independent experiments. P values were determined by one-way ANOVA and student’s *t* test using GraphPad Prism software (* = p<0.05, ** = p<0.001).

As further evidence of the role of IRAK-2 in boosting Th17 development, we found a significantly higher expression of the Th-17-cell associated cytokine IL-22 in IL-1-stimulated CD4 T cells from Why1 than from BL/6 or IRAK-2-/- mice (**Fig. 6D**). IL-23R expression, which is a marker of activated and memory Th17 cells [41], [43], was also significantly higher in anti-CD3/CD28 plus IL-1 stimulated CD4 T cells from Why1 than from BL/6 or IRAK-2-/- mice (**Fig 6E**). Additionally, in Why1 T cells there was significantly higher expression of the Th17 cell lineage-associated transcription factors RORγt (**Fig. 6F**) and AP-1 B-cell activating transcription factor (BATF) (**Fig. 6G**) [17], [44]. Time course analysis revealed an earlier and short-lived induction of Rorγt in comparison with BATF, but at all times both expression levels were higher in Why1 than in BL/6 cells, and were profoundly down-regulated in IRAK-2-/- cells. Interferon regulatory factor 4 (IRF4) and the aryl hydrocarbon receptor (AHR) also play important roles in Th17 cell biology[45], [46], [47], however, there were no significant differences in their expression among the various cell populations (not shown). Likewise, no significant differences were observed in the induction of the Th1 and Th2 cell-associated transcription factors T-bet and Gata-3 (not shown).

For effective induction of responsive genes following TLR/IL-1R stimulation, IRAK family kinases are known to activate a series of downstream signaling events, including NF-κB and certain MAPK family members[48], [49]. To assess the effect of IRAK-2 on these molecular mediators, CD4 T cells were stimulated with IL-1 and the activation of two pathways downstream of the IRAK signaling complex, NF-κB and MAP kinases, were compared. Western blot analysis of phosphorylation levels revealed that in Why1 T cells there was enhanced activation of the IκB kinase (IKK) family member p105 in comparison with BL/6 controls, suggesting increased activation of the NF-κB axis via IL-1 receptor signaling. At the same time, changes in Erk phosphorylation were insignificant, indicating that the MAPK pathway is less affected by the pro-inflammatory IRAK-2 isoform (**Fig. 6H**). Taken together, these results suggest that IL-1-induced Th17 cell polarization via IRAK-2 is associated with increased expression of the transcription factors RORγt and BATF, likely through enhancement of NF-κB activity.

## Discussion

Murine schistosomiasis is a well-established experimental model of a major human infectious disease. Humans as well as mice develop marked differences in disease severity and it is clear that immunopathology is profoundly affected by the host genome. Thus, a greater understanding of its pathogenic mechanisms and underlying genes has widespread implications. Our laboratory has identified several genetic intervals that are associated with severe disease in mice [29], [50], of which some correspond to regions in the human genome that contain the loci *Schistosoma mansoni 1* (*Sm1*) and *Sm2* [51], [52], [53]. Despite these efforts, specific genes that control severe disease have not been identified to date. One reason for this is the genetic redundancy of classical inbred mouse strains, which facilitates genetic analysis of “simple” monogenic and fully penetrant traits. However, greater genetic diversity may be required when investigating traits that are conferred by multiple loci that impart a quantitative contribution to the phenotype. Hence, the limited diversity of classically used strains can make it particularly difficult to identify genes that underlie complex traits, such as those involved in the host response to schistosome infection.

Using the more genetically diverse wild-derived mice as a model, we provide evidence of how genetic mapping of complex traits can be dissected with prior knowledge of the loci or genes identified in relatively simple screens. Previously, we positionally cloned a mutation in the promoter of IRAK-2C that limits the expression of the inhibitory isoform of IRAK-2 in MOLF mice. The outcome of this differential expression is a higher ratio of proinflammatory IRAK-2A relative to the inhibitory isoform IRAK-2C, which in turn leads to an enhanced proinflammatory response in MOLF macrophages[31],[54]. Extending these findings to a physiological setting *in vivo*, we now show that addition of the MOLF *Why1* interval, which contains *Irak2*, markedly increases the levels of IL-17 and the severity of egg-induced hepatic immunopathology in schistosome-infected BL/6 mice. Using a reciprocal approach, we also observed that the deletion of *Irak2* leads to a significant defect in IL-17 production and a marked reduction of immunopathology, thus identifying *Irak2* as the causal gene for this *in vivo* phenotype. The effect of *Irak2* on immunopathology is striking since susceptibility to *S. mansoni* infection is likely conferred by many genes, which have been elusive in previous genetic screens measuring immunopathology as a direct phenotypic read-out. IRAK-2 was not identified in our previous genetic screens in vivo [29], [50]. This is not surprising because these analyses were done in classical inbred mice, which have different levels of pathology but similar IRAK-2 alleles, thus precluding the assessment of the wild-derived IRAK-2 allele in T cell activation during infection. The observed effect of the *Why1* locus and *Irak2* on pathology thus sets an important precedent for how results of a genetic screen *in vitro* can be used for identification of genes influencing complex traits *in vivo*.

IRAK family kinases are central to TLR signaling and a critical factor in innate immunity [55]. Recently, IRAK family kinases have been studied in the adaptive immune response with some discrepancy as to their precise role. IRAK-4 has first been suggested to be an essential factor in TCR induced T cell responses[56]. However, these results have not been confirmed as it was later shown that IRAK-4 is dispensable for normal T cell responses and TCR activity[57], [58]. Here we provide evidence that another IRAK family member, IRAK-2, critically affects T cell biology by regulating the ability of IL-1 to promote Th17 function. Thus, stimulation of T cells with either IL-1 alone, or together with anti-CD3/CD28, resulted in a dramatic increase in IL-17 production by Why1 CD4 T cells compared with BL/6, while IL-17 from IRAK-2-/- T cells was minimal. Stimulation of Why1 CD4 T cells with IL-1 also led to increased activation of the IκB kinase p105, which promotes the degradation of IκB and allows NF-κB to translocate to the nucleus [59]. These observations identify IRAK-2 as a key regulator of Th17 cell biology by enhancing IL-1R signaling through NF-κB activation. This effect of IRAK-2 was specific to Th17 and did not affect IFN-γ production, which is in agreement with recent observations linking NF-κB specifically with IL-17, but not IFN-γ [34]. Our data also show that stimulation with IL-1 in the absence of TCR engagement is sufficient to induce IL-17 production in Why1 T cells, suggesting that their high expression of IRAK-2 is responsible for the increased Th17 responses. However, naive BL/6, Why1 or IRAK-2 -/- T cells did not express the IL-23R or respond to stimulation with IL-23, two hallmarks of activated/memory Th17 cells [18], [20], [41], [42], [60], [61], suggesting that neither one was in a state of activation prior to stimulation. Altogether, these findings imply that the role of IRAK family members in T cell responses is not limited to an effect on TCR signaling, but rather that they can also act via the IL-1R-MyD88 complex to direct Th17 cell responses.

Among several candidate transcription factors, RORγt has been demonstrated to play a central role in Th17 cell differentiation, as its absence significantly impairs IL-17 production [45], [62]. We now show that Why1 CD4 T cells significantly up-regulate RORγt expression following stimulation with IL-1, suggesting that IL-1 *per se* can activate Th17 cells through an IRAK-2 dependent pathway. More recently, BATF was identified as a key transcription factor in Th17 cell differentiation [44], as BATF-deficient mice displayed impaired Th17 cell activity and were resistant to EAE despite normal IL-6 signaling. BATF synergized with RORγt to enhance IL-17 production and sustained RORγt expression in Th17 cells, although the exact nature of their interaction remains to be elucidated [63]. Here we show that BATF expression is significantly enhanced in IL-17-producing Why1 CD4 T cells compared with BL/6 T cells and that this function is IRAK-2 dependent. Interestingly, in our model RORγt expression peaks earlier than BATF (**Fig 6F,G**), suggesting that RORγt may up-regulate BATF during Th17 cell development. Our findings also suggest that BATF functions downstream of the IL-1 receptor thus explaining why BATF-/- mice have a defect in Th17 cell differentiation [44], [63]. IRF4 is another transcription factor involved in Th17 cell differentiation via IL-21[45], [64]. In our system, stimulation with IL-1 resulted in increased IRF4 expression, but levels were not significantly different among BL/6, Why1 or IRAK-2-/- T cells. Likewise, we found no significant differences in the expression of AhR, which has a demonstrated regulatory role in Th17 development and function [47], [65] (data not shown).

As demonstrated in this study, wild-derived IRAK-2 confers on T cells a powerful, TCR-independent hypersensitivity to stimulation with IL-1, which is further amplified in the presence of IL-23. Mechanistically, this is due to a deletion in the IRAK-2C promoter leading to unopposed activation of the main proinflammatory isoform of IRAK-2A. This contrasts with BL/6 mice, in which the inhibitory isoform IRAK-2C is abundantly expressed and up-regulated in response to inflammatory stimuli[31]. Tissue inflammation induces large amounts of IL-1 and IL-23 and it has been suggested that non-antigen specific Th17 cells responding to these stimuli may aggravate tissue damage [26]. In schistosomiasis, IL-1 and IL-23 are highly expressed in MLN and hepatic lesions of high-pathology CBA, but not low-pathology BL/6 mice. Furthermore, dendritic cells derived from the bone marrows of normal CBA mice produce abundant IL-1 and IL-23 in response to stimulation with live schistosome eggs, whereas those from BL/6 mice do not, clearly linking these cytokines with exacerbated disease [28], [30]. Interestingly, IL-1 and IL-23 are also key cytokines for human Th17 cell differentiation [66], and given that humans contain only one proinflammatory isoform of IRAK-2 similar to wild-derived mice [31], it is possible that IRAK-2 may enhance the sensitivity of Th17 cells in a TCR-independent manner and further aggravate immune-mediated tissue damage in human inflammatory diseases.

In summary, using wild-derived mice as a model, we illustrate the first example of a gene controlling severe pathology in murine schistosomiasis, setting an example of how analysis of simple monogenic traits *in vitro* can be applied to complex *in vivo* models of infection or autoimmunity. We used this model to uncover a novel role for IRAK-2 in CD4 T cell signaling via the IL-1 receptor and show that IRAK-2 is a key regulator of IL-1-mediated Th17 cell biology, which may have wide-ranging effects on other Th17 cell-mediated inflammatory diseases.

## Materials and Methods

### Ethics statement

All the animal experiments were performed in accordance with the Guide for the Care and Use of Laboratory Animals of the National Institutes of Health and with the permission of the American Association for the Assessment and Accreditation of Laboratory Animal Care. The protocol was reviewed and approved by the Tufts Medical Center Institutional Animal Care and Use Committee and the Division of Laboratory Animal Medicine (Permit Number: B2009-88).

### Mice, infection and immunization

C57BL/6J, CBA/J and MOLF/Ei (MOLF) mice, 5-8wk old, were purchased from the Jackson Laboratory. Why1 mice were produced as previously described[31]. These congenic mice are homozygous for the MOLF allele selected by marker *D6Mit328* (chromosome 6 at 112.7 Mb) on a BL/6 background. IRAK-2-/- mice were obtained from Dr. Shizuo Akira (Research Institute for Microbial Diseases, Osaka, Japan). Mice were bred and maintained at the Animal Facility at Tufts University School of Medicine in accordance with the American Association for the Assessment and Accreditation of Laboratory Animal Care guidelines. Some mice were infected by i.p. injection with 85 cercariae of *S. mansoni* (Puerto Rico strain) obtained from infected *Biomphalaria galabrata* snails provided by Dr. Fred Lewis (Biomedical Research Institute) through National Institutes of Health/National Institute of Allergy and Infectious Diseases Contract N01-AI-55270. For some experiments, IRAK-2-/-, IRAK-2+/- and BL/6 mice were immunized s.q. with 50 µg of SEA/CFA before and after infection, as previously described[12]. SEA was prepared as previously described[67].

### Assessment of histopathology and egg burden determinations

Formalin-fixed liver samples from 7 week-infected mice were processed for histopathological analysis of 5-µm sections stained with H & E. The extent of granulomatous inflammation around schistosome eggs was measured by computer-assisted morphometric analysis using Image-Pro Plus software (Media Cybernetics) as previously described[29]. At least 15 granulomas were counted per liver. Granuloma size was expressed in square micrometers ± SEM. The schistosome egg load was assessed by counting the number of eggs present in 1 mm^2^ fields of liver tissue in sections stained with hematoxylin/eosin as previously described [68].

### Cell preparations

MLNC suspensions were prepared from individual mice by teasing the lymph nodes in supplemented RPMI 1640 medium containing 10% FCS (Atlanta Biologicals) as previously described[30]. CD4 T cells from MLN or spleens were purified by negative selection using CD4 MACS columns (Miltenyi Biotec) in accordance with manufacturer’s instructions. CD4 T cell purity was >95% by FACS analysis. Liver granuloma cells were isolated as previously described[12].

### Cell cultures and cytokine determinations

Bulk MLNC or GC suspensions (5×10^6^ cells/ml), or purified CD4 T cells (1×10^6^ cells/ml) plus normal irradiated syngeneic splenic APC (4×10^6^ cells/ml), were incubated in the presence or absence of 15 µg/ml SEA for 48hrs. IL-17, IFN-γ, IL-6, TNFα, IL-4, IL-5 and IL-10 protein concentrations in the cell culture supernatants were measured by ELISA using standard cytokines, Abs and protocols from R&D Systems.

### Why1-BL/6 cell cocultures and in vivo CD4 T cell transfers

1×10^6^ purified MLN CD4 T cells from 7 week-infected BL/6 and Why1 mice were cultured *ex vivo* with 4×10^6^ irradiated naïve splenic APCs from BL/6 or Why1 mice for 48 hours in the presence or absence of 15 µg/ml of SEA. IL-17 levels in cell supernatants were measured by ELISA as described. For the cell transfer experiments, BL/6 recipient mice were sublethally irradiated (500 rad) 3 days prior to infection and subsequently injected i.v. with 8×10^6^ naïve splenic CD4 T cells from BL/6 or Why1 donor mice, purified by negative selection as described above. After 7 weeks of infection, IL-17 production by SEA-stimulated bulk MLNC, and by purified MLN CD4 T cells plus irradiated naïve splenic APC, was measured by ELISA as described.

### Quantitative Real-time RT-PCR

Total RNA was isolated from individual samples using TriZol reagent (Invitrogen) as per manufacturers instructions. RNA (1–5 µg) was subjected to DNASE I treatment (Roche) and reverse-transcribed using the high capacity cDNA reverse synthesis kit (Applied Biosystems). Real-time quantitative RT-PCR was performed by SYBR green or Taqman analysis using an ABI 7300 instrument. GAPDH levels were used to normalize the data. Taqman real-time probes for IL-17, IFN-γ, IL-4, IL-12p40, IL-12p35, IL-23p19, IL-22, IL-1β, IL-23R and *batf* were obtained from Applied Biosystems. Primers for SYBR green analysis of *ror*γt were described previously[17]. Using the average mean cycle threshold (Ct) value for GAPDH and the gene of interest for each sample, the equation 1.8 e (Ct GAPDH - Ct gene of interest) ×10^4^ was used to obtain normalized values [69].

### Western blot analysis

1×10^6^ cells CD4 T cells were stimulated with IL-1β (4 ng/ml, R&D Systems) for 0, 5, 10, 20 and 30 minutes followed by lysis on ice in a cytoplasmic lysis buffer (50 mM Tris, pH 8, 150 mM NaCl, 2 mM EDTA, 1% Triton X-100, 1 mM NaVanadate and 10 mM NaF) supplemented with Halt protease inhibitor cocktail (Thermo Fisher Scientific) for 10 min. Lysates were then centrifuged at 13,000 rpm at 4°C for 10 min. Cleared lysates were resolved on a 4-12% gradient Bis-Tris SDS gel (NuPAGE; Invitrogen) and transferred to a nitrocellulose membrane. Rabbit polyclonal antibodies to phosphorylated ERK and p105 were obtained from Cell Signaling Technology. After incubation with specific Abs, chemiluminescence was detected using ECL substrate (Thermo Fisher Scientific).

### Lentiviral transduction

To down-regulate the expression of IRAK-2 in mouse T cells, we used infection with lentiviral particles expressing IRAK-2-targeting shRNA. Lentiviral particles were produced by transfecting (Fugene, Roche) 293-T cells with a plasmid encoding IRAK-2-specific shRNA in the pLKO.1 vector (Open Biosystems clone ID TRCN000022502) together with two other plasmids, pSPAX2 and pMD2.G (Addgene), encoding packaging components of the lentivirus. Supernatants from 293-T cells were harvested on days 2 and 3 after transfection and passed through a 0.22 µm filter.

Naïve CD4 T cells were purified from normal Why1 and BL/6 mouse spleens using the Easysep kit (StemCells). T cells were resuspended to a density of 2×10^6^ cells/ml and plated on 6 well plates that were previously seeded with resident i.p. macrophages from normal BL/6 mice (1.5×10^6^ cells/well). Viral supernatant and medium were added at a 1∶1 ratio for 18 hours. Subsequently, the T cells were washed and allowed to recover for 96 hrs in the presence of macrophages to promote survival. Non-adherent T cells were re-plated at a concentration of 1×10^6^ viable cells/ml for stimulation with anti-CD3/CD28. Cells and supernatants were collected after 48 and 96 hrs.

### Stimulation of naïve CD4 T cells

Naïve CD4 T cells were incubated in either 96 well plates (3.5×10^5^ cells/ml) for ELISA detection, or 48 well plates (1×10^6^ cells/ml) for real-time analysis in triplicates, and stimulated with anti-CD3/CD28 coated beads (3×10^5^, Dynal) together with rIL-1β, at indicated concentrations, and rIL-23 (20 ng/ml). For ELISA, cell culture supernatants were collected after 4 days and analyzed for IL-17 as described. For real-time PCR, cells were collected at 0, 2, 24, 48 and 96 hrs in Trizol reagent and assayed as described.

### Statistical analyses

ANOVA and Student’s *t* tests were used to determine the statistical significance of the differences between groups and were calculated with GraphPad Prism.

### Accession numbers

*Il17a* (Mouse Genome Informatics:107364), *Ifng* (MGI:107656), *Il6*(MGI:96559), *Tnf* (MGI:104798), *Il4* (MGI:96556), *Il5* (MGI:96557), *Il10* (MGI:96537), *Irak2* (MGI:2429603), *Rorc* (MGI:104856), *Batf* (MGI:1859147), *Irf4* (MGI:1096873), *Ahr* (MGI:105043).
